# Akt modes of stem cell regulation: more than meets the eye?

**DOI:** 10.15190/d.2013.8

**Published:** 2013-12-31

**Authors:** Enzo Calautti

**Affiliations:** University of Turin, Department of Molecular Biotechnology and Health Sciences, Turin, Italy

**Keywords:** Akt in stem cell biology, Akt signaling in stem cell self-renewal and differentiation, Akt signaling in metabolism and stress

## Abstract

Akt signaling regulates many cellular functions that are essential for the proper balance between self-renewal and differentiation of tissue-specific and embryonic stem cells (SCs). However, the roles of Akt and its downstream signaling in SC regulation are rather complex, as Akt activation can either promote SC self-renewal or depletion in a context-dependent manner. In this review we have evidenced three "modes" of Akt-dependent SC regulation, which can be exemplified by three different SC types. In particular, we will discuss: 1) the integration of Akt signaling within the "core" SC signaling circuitry in the maintenance of SC self-renewal and pluripotency (embryonic SCs); 2) quantitative changes in Akt signaling in SC metabolic activity and exit from quiescence (hematopoietic SCs); 3) qualitative changes of Akt signaling in SC regulation: signaling compartment-talization and isoform-specific functions of Akt proteins in SC self-renewal and differentiation (limbal-corneal keratinocyte SCs). These diverse modes of action are not to be intended as mutually exclusive. Rather, it is likely that Akt proteins participate with multiple parallel mechanisms to regulation of the same SC type. We propose that under specific circumstances dictated by distinct developmental stages, differentiation programs or tissue culture conditions, one mode of Akt action prevails over the others in determining SC fates.

## Introduction

A stem cell (SC) is defined as an undifferentiated, long-lived proliferating cell with the capacity to self-renew and to generate progenies of cells primed to differentiate in one or more cell types^1^. With this dual function (or “stemness”), SCs allow the generation of tissues during development by building their basic cellular blocks, and their maintenance throughout lifetime by counter-balancing the continuous loss of differentiated or damaged cells.

The search of a common set of genes (the “stemness” genes) among embryonic, neural and hematopoietic SCs has failed to reveal a gene expression signature accounting for the SC status in all cell types analyzed^2,3^, and the emerging view is that in different contexts, stemness is maintained by largely independent cellular programs, each one suited to couple SC self-renewal and differentiation abilities with distinct developmental stages, tissue differentiation programs and cell turnover rates^4-6^.

In fact, besides playing their defining roles, SCs are first of all cells, and must regulate fundamental biological functions such as the ability to survive, to divide and migrate, to establish contacts with neighboring cells and the surrounding environment (niche), to regulate metabolism in response to nutrients availability, and to respond to a variety of external cues and cellular stresses. In particular, cell-protective mechanisms coincide with many of the conserved signaling pathways that regulate SC self-renewal and differentiation^7^. Therefore, maintenance of functional SC pools during the organism lifespan requires integration of SC-specific signaling mechanisms (the “core” SC signaling circuitries) with other key cellular programs, fitted to maintain “stemness” and tissue homeostasis in the context in which SCs operate.

Consistent with a role of SCs in aging^8^, it seems conceivable that stemness arose during evolution in parallel with the emergence of long-lived multicellular organisms, as a strategy to prolong lifespan via elimination and replacement of differentiated or damaged cells in increasingly complex tissues. One conserved molecular machinery coupling the cell responses to stresses and nutrients availability with the regulation of organismal aging and SC biology is the PI3K/Akt/FOXO/mTOR signaling network^9,10^. The most evolutionary conserved function of Akt signaling is the control of energy metabolism, which in mammalian cells is coupled to the ability to inhibit apoptosis and promote cell cycle progression (for review see^11,12^). Attenuation of the insulin/IGF-1-like (IIS) metabolic pathway as well as caloric restriction extends the lifespan of nematodes, fruit flies and mammals^8,9,13,14^. Effectors of lifespan extension induced by inhibition of IIS are the forkhead box O (FOXO) family of transcription factors, which are under the negative control of the IIS/PI3K/Akt pathway^13,15-18^. Individual or combined ablation of FOXO genes in mice leads to SCs depletion in various lineages^19-21^. Inhibition of mTORC1, a signaling complex activated by IIS/Akt signaling and nutrients^22^, also extends the lifespan of model organisms^23-25^, and mTORC1 pharmacological inhibition by rapamycin enhances SC functions in several contexts^26-29^. Moreover, a conserved role of FOXOs in limiting mTORC1 signaling by inducing the expression of mTORC1-inhibitory proteins has also been established^30-32^. However, in spite of the conserved functions of these Akt effectors pathways in organism lifespan and SC maintenance, the consequences of Akt activation in different SC types range from enhanced SC self-renewal^33,34^ to premature SC exhaustion^35,36^. In the attempt to reconcile these apparent paradoxes, we will provide here a simplified view of three diverse modalities by which Akt signaling regulates SC self-renewal and differentiation, exemplified by three different SC types.

## Akt proteins and activation

Akt/PKB signaling is carried out by three homologous serine/threonine protein kinases (Akt1/PKBα, Akt2/PKBβ and Akt3/PKBγ) encoded by separate genes^37,38^. In response to a plethora of extracellular signals Akt kinases regulate fundamental cellular functions such as growth, survival, differentiation and energy metabolism^39^. Activation of Akt kinases normally occurs downstream of phosphoinositide-3 kinases (PI3K). Full enzymatic activation is achieved through a sequential process. Akt proteins are first recruited to the plasma membrane through their amino-terminal pleckstrin homology (PH) domains at sites enriched of PI3K lipid products (PI(3,4,5)P3 or PI(3,4)P2). At the plasma membrane, Akt proteins are phosphorylated at two conserved residues, the first one within the T-loop of the catalytic domain (Thr308 in Akt1) by the phosphoinositide-dependent kinase 1 (PDK1), and the second one within the carboxy-terminal hydrophobic motif (Ser473 in Akt1) by the mammalian target of rapamycin complex 2 (mTORC2)^40-42^, albeit other protein kinases have also been found capable of phosphorylating the same Ser residue^43^**. **Although phosphorylation of Akt at both regulatory residues is critical for full kinase activation, it has been shown that in the absence of Ser473 phosphorylation, Akt is still capable to phosphorylate some, but not all its substrates^41^.

Akt proteins are localized to the plasma membrane following activation, and once activated, they distribute throughout different cell compartments including the nucleus, whereby they phosphorylate a multitude of substrates to regulate diverse cellular functions^39^. Akt kinases are inactivated by protein phosphatases such as the protein phosphatase 2A (PP2A), which dephosphorylates the regulatory Thr residue within the kinase domain^44^, and the family of plekstrin homology domain leucine-reach repeat protein phosphatases (PHLPP1 and PHLPP2) that dephosphorylate the Ser residue of the hydrophobic motif of Akt kinases in an isoform-specific manner^45^.

The overall PI3K/Akt signaling activity is negatively regulated by the tumor suppressor PTEN that dephosphorylates the PI3K lipid products, and by activation of the Akt downstream effector mTORC1, which in turn induces negative feedback mechanisms that limit PI3K activation (for review see^46,47^. Hyperactivated Akt is a common feature of many human cancers^39^ since, at least in part, it both provides protection from apoptosis and promotes uncontrolled cell-cycle progression^48^.

Studies of Akt isoform-specific knockout mice have recently suggested that besides playing redundant roles in cellular signaling, Akt isoenzymes have also specific biological functions^49^. Akt1 knockout mice have growth retardation^50^ and Akt1-null cells display higher rates of apoptosis, indicating a critical role for Akt1 in cell survival^50,51^. Akt2 knockout mice develop a type 2 diabetes-like phenotype, and cells derived from those mice show impaired glucose utilization^52,53^, suggesting a key role for Akt2 in the regulation of glucose homeostasis. Consistently, protein interaction studies have recently confirmed such a metabolic function for Akt2^54^. A role for Akt3 in brain development has also been proposed since Akt3 knockout mice display decreased brain size^55^. Moreover, during tumor development, Akt1 and Akt2 often act in a complementary opposing manner^37,38^. Specific gain of function of Akt2 promotes migration and invasion in breast cancer epithelial cells, whereas Akt1 is mostly involved in cell proliferation and growth^56^. In addition, Akt2 induces epithelial to mesenchymal transition^57,58^, a process involved in *de novo* formation of breast cancer SCs^59^. Intriguingly, the differential roles of Akt isoforms in breast cancer SC have been found associated with a differential regulation of microRNA of the miR200 family by individual Akt isoforms^60^. One outstanding issue is how such signaling specificity is achieved in spite of the high structural homology of Akt isoforms.

## Signals downstream of Akt protein kinases in SC regulation: life span, metabolism and stress

Key effectors of Akt signaling in SC regulation are the mTORC1 signaling complex, positively regulated by Akt signaling^22^, and the FOXO family of transcription factors, which are directly inhibited by Akt-dependent phosphorylation^15-18^.****A persistently elevated mTORC1 activity causes an initial expansion followed by a later decline of several adult SC types^26,61^, whereas individual or combined ablation of FOXO genes in mice leads to SC depletion in various lineages^19-21^. In both fly and mammalian cells, FOXOs can restrain mTORC1 activity^30,31,62,63^, and this regulatory network has been recently implicated in stratified epithelial SC regulation^64^. Members of the FOXO class of transcription factors (FOXO1, -3, -4 and -6) regulate diverse gene expression programs and affect many cellular processes, including cell cycle regulation, cell survival and metabolism^65,66^. Two evolutionarily conserved pathways regulate FOXO activity: in the presence of growth factors, FOXOs are negatively regulated by the canonical IIS signaling pathway through PI3K and AKT^65^; FOXOs are activated instead in the presence of oxidative stress through Jun N-terminal kinase (JNK)^67^. Additionally, many other signaling pathways and multiple post-translational modifications modulate FOXO activity^65-69^. All FOXO family members bind to the consensus sequence (5′-TTGTTTAC-3′), and therefore are thought to act redundantly, although selective functions of specific FOXO members have also been described. The FOXO homologue in Caenorhabditis elegans, daf-16, was genetically linked to daf-2, which encodes an insulin type receptor, and shown to mediate the lifespan extension resulting from daf-2 loss, thus providing evidence of a crucial role of the PI3K/Akt/FOXO signaling axis in lifespan control^9^. FOXOs integrate a multitude of signals from pathways that are sensitive to environmental changes, and the emerging view is that these transcription factors behave as key regulators of tissue homeostasis under stressful conditions^10^.

## 1) Integration of Akt signaling within the “core” pluripotency signaling network in the mainte-nance of SC self-renewal and pluripotency (embryonic SCs)

Murine embryonic SCs (mES cells) are cell lines derived from the inner cell mass of the mouse blastocyst^70,71^ and can be indefinitely expanded in an undifferentiated, pluripotent state by the addition of leukemia inhibitory factor (LIF) to the culture medium. ES cells need to tightly balance their gene expression program to prevent differentiation while maintaining pluripotency, which is executed by a gene network that is centered around the transcription factors OCT4, SOX2 and NANOG^72^. In addition to activating the Jack/Stat and the MEK/ERK pathways, LIF also engages the PI3K/Akt pathway in mES cells^73^. In these cells, ectopic expression of a myristoylated, active form of Akt is sufficient to maintain the pluripotent, undifferentiated cell phenotype in the absence of LIF^74^. Importantly, the effects of active Akt were found to be reversible, since deletion of the transgene restored both LIF dependence and pluripotent cell differentiation. It has been further demonstrated that LIF to Akt signaling is critical for maintaining elevated levels of ES cell-specific transcription factors such as Tbx3 and Nanog^75,76^, the latter being an essential determinant of the “naïve” ES cell pluripotent state^77^. Moreover, Akt has also been reported to phosphorylate and enhance the transcriptional activity of Sox2^78^, another key factor in both ES cell pluripotency and reprogramming of somatic cells to induced-pluripotent stem cells (iPS).

Differently from mES cells, human ES (hES) cells do not depend on LIF signaling for self-renewal but rely on different combinations of exogenous factors including Activin, an agonist of Smad/2/3 signaling (for review see^79^). In hES cells, Activin favors pluripotency by promoting Nanog expression^30,80^ but also drives mesodermal differentiation when PI3K/Akt signaling is low^81^. hES cells can be maintained in a pluripotent, undifferentiated state under defined culture conditions requiring only three exogenous factors^82^, Activin, and two potent activators of PI3K/Akt signaling, heregulin and Igf-1. It has been shown that omission of extracellular Akt agonists or inhibition of Akt intracellular signaling promotes mesodermal differentiation. Since Activin/Smad2/3 signaling is also essential for hES self-renewal, it has been demonstrated that inhibition of PI3K/Akt activity skews the functions of Smads from self-renewal to differentiation. In particular, Akt activity, by restraining ERK and Wnt-β catenin signaling, on one hand maintains Smad-dependent Nanog expression, and on the other hand prevents Smads from activating mesodermal genes^82^.

Interestingly, proliferating ES cells have a metabolic signature that is reminiscent of that of quiescent hematopoietic SCs (HSCs). ES cells have a low mitochondrial mass compared to their differentiating cell progenies and cell differentiation parallels with a switch from glycolysis to a robust increase in mitochondrial oxidative activity^83^.

Since one of the most conserved effects of Akt activation is the increase in cellular glucose uptake and stimulation of oxidative metabolism, which are mediated in large part by inhibition of FOXOs and activation of mTORC1 signaling, how ES cells maintain high Akt activity to promote self-renewal, and at the same time maintain low oxidative rates is currently unclear. This would suggest that Akt proteins, in ES cells, are decoupled from some of their key signaling effectors in cell metabolism. Intriguingly, FOXO1 has been recently shown to be necessary for ES cell pluripotency, and to directly regulate OCT4 and SOX2 expression and functions in an Akt-independent manner^84^. How FOXO1 can be transcriptionally activated in these rapidly dividing cells, and how its activity is directed towards OCT4 and SOX2 deserve future investigation.

In summary, increasing experimental evidence indicates that in both mES and hES, Akt signaling participates to the “core” pluripotency network by impinging on Nanog expression, and that gains of Akt activity promote pluripotency and self-renewal at the expenses of differentiation, in a manner that is decoupled from activation of Akt metabolic effectors. However, the identities of the direct Akt effectors in this context are still unknown.

## Differential roles of Akt signaling in tissue-specific SCs

In tissue-specific SCs, the long-term consequences of sustained Akt activation, such as that induced by PTEN deletion, range from enhanced SC expansion and maintenance like in neuronal and mammary gland progenitor cells^33,34^, to SC depletion in the hematopoietic system^35,85^. The mechanisms underlying these opposite roles of Akt signaling in different SC types are still unclear, and may include differential ability of Akt protein kinases to phosphorylate a subset of substrates under specific conditions, the strength and duration of Akt activity, the onset of negative feedbacks mechanisms, as well as cross talks with other key SC regulatory signaling pathways (Notch, Wnts, BMP, Activin/TGF-β), ultimately determining the choice of a SCs towards a specific fate.

## 2) Akt regulation of SC metabolism and quiescence (Hematopoietic Stem Cells)

Hematopoietic SCs (HSCs) are cells that give rise to the cellular constituents of blood for life-long. Although SC quiescence is not a general prerequisite of all stem cell types^86,87^, multipotent HSCs are typically slow cycling, and become activated to generate committed progenitor cells that replenish all the differentiated blood cell lineages. HSCs reside normally in hypoxic niches in the bone barrow^88^, and relatively low oxygen levels promote SC quiescence^89,90^ and glycolytic metabolism at the expenses of mitochondrial oxidative metabolism (reviewed by^83,91^), whereas physiological increases in reactive-oxygen species (ROS) are coupled with the differentiation switch^20,36,92,93^. Proper levels of ROS and maintenance of reversible quiescence play thus key roles in long-term HSC maintenance, since virtually every genetic or environmental manipulation permanently altering these parameters usually results in hematopoiesis abnormalities, including leukemia (reviewed by^94^).

Molecules downstream of Akt signaling, including FOXO transcription factors and the mTORC1 signaling complexes are critical mediators of the cell metabolic responses to changes in nutrients, growth factors and oxygen levels. Akt activation, by favoring glucose uptake metabolism, is a potent activator of glucose uptake and mitochondrial oxidative metabolism. Moreover, mTORC1 signaling, which is negatively regulated by hypoxic conditions and positively by Akt, signaling, stimulates mitochondrial biogenesis (reviewed by^95^). The transcriptional activity of FOXO family members is stimulated by oxidative stress, and inhibited by Akt activation in response to growth factors, and FOXO target genes are crucial mediators of HSC quiescence, and resistance to oxidative stress.

In the hematopoietic system, PI3K/Akt/FOXO signaling plays critical roles in both HSC maintenance and lineage development^96^. Several hematopoietic growth factors and cytokines, such as erythropoietin, thrombopoietin, Stem Cell Factor (SCF, c-kit-ligand), Flt3 ligand, activate the PI3K/Akt pathway. FOXOs have a crucial role in several types of adult stem cells and as this role seems to be evolutionarily conserved, since they also regulates SC maintenance in Hydra vulgaris^97^. In mHSCs they favor SC maintenance and self-renewal ability^19,20,98^. In addition, FOXOs may protect HSCs from cell death by mediating resistance to physiological oxidative stress thorough catalase and superoxide dismutase. Indeed, whereas targeted deletion of FOXO in HSCs results in HSC exhaustion with concomitant increase in cellular ROS, treatment with an antioxidative agent could partially rescue this phenotype^20,98^. This role of FOXOs in maintaining the SC population over time also occurs in neural SCs^21,99^. Consistent with the suppression of PI3K/Akt signaling observed in HSCs, mTORC1 is downregulated in these cells and studies on TSC-1 knockdown have shown that mTORC1 inhibition is essential for HSCs quiescence^98,100,101^. Besides, HSCs functions decline with age and this is reportedly due to increased mTORC1 activity^26^.

Moreover, several developmental signaling pathways that also regulate adult hematopoiesis (Wnts, Notch and BMP)^102^, can potentially cross talk with the PI3K/Akt pathway at multiple levels. Quantitative abnormalities in Akt signaling have a deep impact on HSC biology. For instance, in murine HSCs, both conditional deletion of PTEN^35,85^ and overexpression of a constitutively activated myrAkt^103^ cause loss of quiescence, transient expansion of HSCs, followed by long-term SC decline. Importantly, in both experimental settings, the exhaustion of HSCs is paralleled by the development of leukemia-like hematological disorders. On the other hand, the combined deletion of Akt1 and Akt2, the two main Akt isoforms expressed by hematopoietic cells, results in exaggerated SC quiescence, reduced ROS production, and impaired cell differentiation, consistent with the notion that physiological levels of ROS are involved in the switch between HSC quiescence and differentiation^104^. Serial HSC transplantation was impaired by Akt1/Akt2 deficiency, albeit individual deletion of either Akt isoform had little or no effect. Overall, these studies indicate that HSCs are extremely sensitive to the overall levels of Akt signaling output, and whereas normal Akt signaling is required to balance SC self- renewal with normal ROS production and differentiation, either too much or too little Akt activity eventually result in SC failure.

Several transcription factors are necessary for HSC self-renewal, among which GATA2, GFI1, TEL, JUNB, SOX17 and PU.1^102^ . Although phosphorylation of GATA2 by Akt has been reported to regulate adipose cell differentiation^105^, it is currently unknown whether a similar mechanism is also involved in HSCs self-renewal and differentiation. Inhibition of PI3K/Akt/ mTORC1 signaling also inhibits T cell differentiation, and prevents downregulation of the GFI1 transcription factor^106^. However, whether or not Akt signaling plays a direct role in the regulation of the core HSC transcriptional network remains to be established.

On the other hand, there is accumulating evidence that Akt directly participates to the epigenetic regulation of HSCs by phosphorylating Bmi1, one key component of the Polycomb Repressor Complex 1 (PRC1), at Ser 316, thereby favoring de-repression of the Ink4a-Arf locus, which encodes for p16/Ink4a and p19/Arf proteins that promote cell cycle arrest and cell senescence. Importantly, ablation of Bmi1 in mice also leads to failure of several SC types including HSCs^107^, and more in general, defects in either PRC1 or PRC2 functions hamper SC self-renewal in several cell types, including HSCs^108^. Akt also phosphory-lates the EZH2 catalytic component of PRC2, thereby counteracting its repressive activity on gene expression^109^. Therefore, under conditions of persistently elevated Akt activation, such as PTEN loss, HSC exhaustion may depend in part on cell senescence favored by loss of Polycomb repressive activity at the Ink4a-Arf locus.

It has been shown that mTORC1 inhibition by rapamycin rescues the exhaustion of normal HSCs and prevents the onset of leukemia following PTEN loss *in vivo*^35^. Consistently, conditional deletion of the mTORC1 negative regulator TSC1 in murine HSCs provokes loss of SC quiescence, increased mitochondrial biogenesis and ROS production, with subsequent failure of hematopoiesis and HSC self-renewal^101^. Importantly, the effects of TSC1 deletion were rescued by treatment of cells with ROS antagonists. Since Akt phosphorylates TSC1/ TSC2 complexes to release their inhibitory activity on mTORC1, these data strongly suggest that the PTEN/Akt/mTORC1 axis normally plays important roles in the normal balance between HSC self-renewal and differentiation and in PTEN/Akt-induced HSC exhaustion and leukemogenesis (for review see ^95^).

Consistently, in murine HSCs, conditional deletion of Raptor, an essential component of mTORC1, severely hampers hematopoiesis leading to pancytopenia, splenomegaly, extramedullary hematopoiesis and abnormalities in differentiation^110^. Raptor-deficient HSCs display abnormalities in transplantation assays and impaired regenerative potential upon sub lethal irradiation. Notably, Raptor depletion also halts leukemic progression in PTEN-deficient HSCs.

On the other hand, the conditional deletion of Rictor (an essential component of mTORC2) in adult mice has no effects on basal hematopoiesis, but prevents both SC exhaustion and leukemogenesis induced by PTEN loss^111^. These data are consistent with a model in which mTORC1 plays key roles in both normal SC maintenance and Akt-driven SC depletion, while mTORC2, by favoring Akt activation from upstream, becomes a limiting factor in cellular processes that rely on maximal Akt signaling outputs, such as those causing HSC depletion and leukemogenesis.

Altogether, these studies provide compelling evidence that mTORC1 signaling activity is essential for normal hematopoiesis and represents a driving force behind HSC depletion and leukemia progression downstream of the PTEN/Akt axis.

Thus, mTORC2 and mTORC1 appear to represent respectively an essential regulator and a key effector of Akt activation in both HSC depletion and leukemogenesis induced by PTEN loss. Does this mean that FOXOs, established regulators of HSC maintenance and direct Akt targets, do not play a role in this process? Sustained Akt activation downstream of PTEN loss is expected to lead to FOXO inactivation, and since FOXO loss of function *per se* drives HSC depletion, it is conceivable that also in the context of PTEN loss, Akt-dependent FOXO inactivation may contribute to SC depletion. Intriguingly, several direct FOXO target genes including sestrin3,^32,112^ and the direct TORC1 inhibitor TSC1 have the potential to restrain mTORC1 signaling via different mechanisms. Notably, this FOXO-dependent mTORC1 inhibition has been shown to take place under different experimental conditions and is though to represent a conserved cellular response to cellular stress conditions between flies to mammals^113^. Moreover, we have recently demonstrated that in cultured human limbal keratinocyte SCs, the Akt2/FOXO/mTORC1 signaling axis is a critical determinant of epithelial stem cell exhaustion, at least in culture^64^, suggesting that this cross talk may be important also in the regulation of other SC types in which robust Akt signaling to FOXO plays a role in SC depletion. Since both FOXOs and TSC1 deletion in HSCs result in SC depletion and exaggerated ROS accumulation, and in both cases SC depletion is counteracted by ROS scavengers, this further suggests a convergence of FOXO and mTORC1 signaling on similar cellular process in HSC regulation. Therefore, it would be interesting to test whether or not the HSC decline induced by loss of FOXOs can be rescued in part by rapamycin treatment, like in the case of PTEN loss. Should this not be the case, this would suggest that FOXOs might become desensitized to Akt inhibition due to activation of other cellular pathways. It has been reported that in certain cellular contexts, transcriptional activation of FOXOs by JNK upon stress including oxidative damage, dominates over the ability of Akt to inactivate FOXOs^67^. Clarifying the role of FOXOs in Akt-induce SC depletion and leukemogenesis is of key therapeutic relevance, since it has been recently reported that in Acute Myeloid Leukemia (AML) cells, FOXOs favors the maintenance of cells with the properties of leukemia-initiating cells. Unexpectedly, either Akt activation or FOXO inhibition induce apoptosis and promote myeloid differentiation of AML cells, reverting the established paradigm of Akt behaving as an oncogene, and FOXOs as tumor suppressors. Interestingly, AML cells that escape from Akt-induced FOXO inactivation or loss of FOXO expression, become sensitive to JNK inhibitors, suggesting that JNK activation probably favors the selection of AML cells capable to withstand the loss of FOXOs, providing a rationale for the employment of JNK inhibitors in antileukemic therapy. Importantly, quiescence is a property shared by normal multipotent HSCs and Leukemia–initiating cells.

## 3) Akt signaling selectivity in SC regulation: subcellular compartmentalization and isoform-specific functions of Akt proteins in human limbal keratinocyte stem cells (hLSCs)

The cornea is the most important light refracting structure of the eye, and the integrity of its stratified epithelium is key for visual acuity. In adult humans, the self-renewal of the corneal epithelium relies on keratinocyte SC populations that reside in the basal epithelial layer of the limbus, the narrow transitional zone at the boundaries between the cornea and the bulbar conjunctiva^114-121^. Pathological conditions leading to loss of limbus integrity result in severe visual impairment or blindness. The similarities between human limbal- and epidermal keratinocytes, which have been extensively employed in cellular therapies for treating burn patients, have allowed the establishment of culture conditions apt to propagate hLSCs *ex vivo* for use in therapeutic transplants^17-19,121-123^. Culture of human limbal keratinocytes (LKs) in the presence of a fibroblast feeder layer^124^ plus additional factors, including fetal bovine serum, Insulin and Epidermal Growth Factor, originate three types of colonies, namely, holoclones, meroclones and paraclones^117,122^, of which holoclones possess all the defining features of cultured SCs such as long-term self-renewal and the capacity to regenerate tissue *in vivo*^125,126^. We have recently explored the role of Akt signaling in the biology of hLSCs^64^.

We have found that in human corneal tissue, the distribution of active Akt proteins nearly overlaps with that of the putative LSCs marker p63, which, under homeostatic conditions, is confined to the basal layer of the limbal epithelium^127,128^. Moreover, few differentiating cells moving to the suprabasal differentiated cell layers, expressing low levels of p63, also display high levels of Akt activation.

The progenies of cultured human primary limbal keratinocyte holoclones have also higher Akt activity as compared to those generated SC-depleted clones. We found that a transient inhibition of PI3K or Akt activity impairs the clonogenic potential of the progenies of treated cells in spite of having little or no effects on the cell cycle, suggesting that PI3K to Akt signaling has important roles in LSC self-renewal.

By investigating the roles of Akt1 and Akt2, the two Akt isoforms expressed in LKs, we found that whereas Akt1 silencing anticipated SC exhaustion, Akt2 depletion had an unexpected effect on cultured LSCs self-renewal ability, since it significantly enhanced both clonogenic potential and lifespan of cultured cells and interfered with differentiation and senescence cellular programs. Consistently, in Akt2-deficient mice, the amounts of progenitor cells expressing the p63 limbal stem cell marker is increased, suggesting that Akt2 deficiency also affects LSC biology in vivo.

Our data indicated that signaling specificity rather than drops in global Akt activity underlies the SC phenotype of Akt2-deficient LKs, since their sustained self-renewal ability can be selectively reverted by ectopic re-expression of a normal Akt2 protein, but not by comparable levels of either kinase-dead Akt2 or wild-type Akt1. Akt2 depletion selectively attenuates growth factors signaling to both FOXOs and mTORC1, albeit in a hierarchical manner, whereby an increase in FOXOs expression and transcriptional activity leads to mTORC1 inhibition by promoting the expression of its negative regulator TSC1. Thus, our work provides direct evidence that the conserved FOXO/mTORC1 axis participates to the SC regulation, and that at least in this particular SC type, one single hit (Akt2 depletion) is sufficient to enhance FOXO functions and to restrain mTORC1 activity.

Our work has indicated that the mechanistic bases of the differential effects of Akt1 and Akt2 reside mostly in their differential subcellular distribution; whereas Akt2 protein is both nuclear and cytosolic, Akt1 is found almost exclusively in the cytosol, but when its expression is enforced in the nuclear compartment via a nuclear localization signal peptide, Akt1 acquires the ability to revert the SC phenotype of Akt2-deficient cells and to phosphorylate FOXO molecules. These data suggest that is not Akt2 per se, but rather its nuclear distribution that drives LSC depletion.

Our study opens up many additional questions that need to be addressed to shed light on the complexity of Akt functions in SC regulation. Does the effect of Akt2 depletion reflect the well-established role of Akt2 in metabolism control? Consistent with the notion that oxidative stress represents one major determinant of cultured cell senescence^29^, our unpublished data indicate that Akt2-depleted LSCs display enhanced resistance to oxidative damage. It is worth remembering that the ocular surface is highly exposed to oxidative stress^7^, and that corneal epithelial cells have large stores of glycogen that is used as a primary energy source. Akt signaling has been recently shown to couple hypoxic signaling with glycogen metabolism in the limbal-corneal tissue^129^. It would be interesting to investigate how individual Akt isoforms regulate these functions in the context of LSC self-renewal and differentiation.

Another key issue is to better understand the molecular determinants of Akt proteins nuclear distribution. This aspect of research seems particularly relevant for SC biology since it would explain how Akt proteins are targeted to transcriptionally active FOXO proteins as well as to other nuclear targets potentially involved in SC regulation. However, the molecular determinants of Akt isoforms nuclear distribution are still poorly understood, and the possible underlying mechanisms include phosphorylation, ubiquitylation and interaction with specific protein partners^38,130^.

How nuclear Akt signaling integrates in the core transcriptional programs and epigenetic regulation of LSCs and other SC types regulated by similar control mechanisms (such as for instance epidermal keratinocyte SCs)? Our work suggests a tight correlation between PI3K/Akt/FOXO signaling and p63 regulation, and Akt2-depleted cells express high levels of ΔNp63α the p63 isoform most involved in the regulation epithelial SC proliferative potential^131^. Previous work has indicated that PI3K signaling positively regulates p63 expression at the transcriptional level^132^. Moreover, the IIS/FOXO pathway has been recently shown to regulate p63- dependent gene expression in the establishment of epidermal stratification during mouse development^133^. Our work suggests that p63 may be both positively and negatively regulated by Akt signaling, and these findings may have translational implications in regenerative medicine, since high levels of ΔNp63α expression correlate with the success of cultured LSC grafts in therapeutic settings^119,126^.

Notably, our studies indicating that Akt2 opposes normal epithelial SC functions are apparently at odds with the notion that in a human immortalized breast cell line, Akt2 favors *de novo* formation of cancer SCs^57-60^. Hence, we believe that caution should be taken when interpreting the biological function of Akt signaling in normal epithelial cells or in immortalized and/or transformed cell lines that already evaded fail-safe mechanisms of tumor suppression.

**Figure 1:**
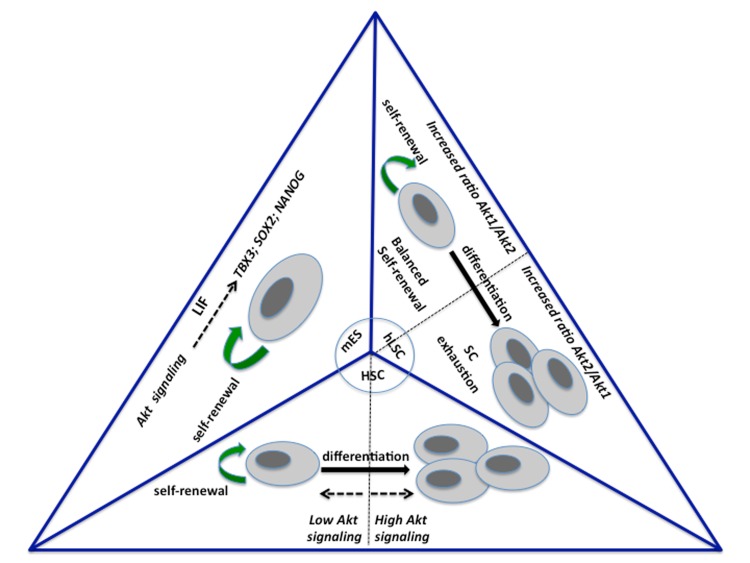
Schematic representation of the three “modes” of Akt signaling described in the main text a) in mouse embryonic stem cells (mES), Akt signaling favors self-renewal and pluripotency at the expenses of cell differentiation (not shown); b) in hematopoietic stem cells (HSC), quantitative changes in Akt signaling outputs determine different SC fates: normal Akt signaling favors self-renewal (low Akt signaling), while an increase of activity (high Akt signaling) favors exit from quiescence and commitment to differentiation c) in human limbal keratinocyte stem cells (hLSC), a different ratio between Akt isoforms activity in discrete subcellular compartments shifts the balance between SC self-renewal and differentiation.

## Conclusions

PI3K/Akt signaling has been shown to either promote or oppose SC maintenance. Although a large number of studies indicate a close link between metabolic pathways and SC regulation by Akt kinases, we still have a very limited understanding about the integration of these processes by Akt family members. In this review, we have provided three different examples of modalities (Figure 1) by which Akt signaling regulates SCs in three different biological contexts. These diverse “modes” of Akt action are not to be intended as mutually exclusive. On the contrary, it is likely that Akt proteins participate with multiple parallel mechanisms to regulation of the same SC type. We propose that under specific circumstances dictated by distinct developmental stages, differentiation programs or tissue culture conditions, one “mode” of Akt action prevails over the others in determining the SC fate.

- **Akt and its downstream signaling effector molecules play key roles in SC self-renewal and differentiation, in a context dependent manner **

## Future AIMS and Open QUESTIONS:

- **Define the interconnections between Akt signaling that govern metabolic/stress events and stem cell specific functions, namely self-renewal and differentiation**

- **Determine specific roles of Akt isoforms in stem cell regulation: differential response to stimuli, specific subcellular distribution and substrate specificity**

- **Define the functions of Akt isoforms and their downstream targets; this is crucial for implement-tation of PI3K/Akt pharmacological treatments into the clinic, both in disease and aging, as well as for producing functional SCs for regenerative medicine**
- **Manipulate metabolic and stress pathways in order to implement normal stem cell therapies and/or target cancer stem cells**
